# Partnering with Communities to Understand Social Determinants of Health (SDoH) Impacts on Access to Shared Micromobility

**DOI:** 10.3390/ijerph21111488

**Published:** 2024-11-08

**Authors:** Elizabeth K. McClain, Kaitlynn Walker, Ganesh Kumar, Ashley Bright, Klare Aziz, Ann W. Banchoff, Zakaria N. Doueiri, Abby C. King, Suman K. Mitra

**Affiliations:** 1 Department of Medical Education, Arkansas College of Osteopathic Medicine, Fort Smith, AR 72916, USA; 2 Arkansas College of Osteopathic Medicine, Fort Smith, AR 72916, USA; kwalker@achehealth.edu (K.W.); gakumar@achehealth.edu (G.K.); abright@achehealth.edu (A.B.); kaziz@achehealth.edu (K.A.); 3 Department of Epidemiology & Population Health, Stanford University School of Medicine, Palo Alto, CA 94304, USA; banchoff@stanford.edu (A.W.B.); doueiriz@stanford.edu (Z.N.D.); king@stanford.edu (A.C.K.); 4 Department of Medicine (Stanford Prevention Research Center), Stanford University School of Medicine, Palo Alto, CA 94304, USA; 5 Department of Civil Engineering, University of Arkansas, Fayetteville, AR 72701, USA; skmitra@uark.edu

**Keywords:** shared micromobility, social determinants of health, photovoice, citizen science/Our Voice, underrepresented/vulnerable community

## Abstract

This study explored the facilitators and barriers of community bike share use in a mid-sized city with high incidence of poverty and racial diversity using a community-based participatory action research (CBPAR) photovoice framework with the Stanford *Our Voice* (OV) Discovery Tool digital application. Community members participated in one of three community citizen science walks with follow up focus groups facilitated by osteopathic medical student researcher to address “What makes it easy or hard to ride a bike using the bike share?” Twenty-seven diverse community members partnered with four osteopathic medical students exploring vulnerable individuals’ lived experiences, beliefs/understanding of the Social Determinants of Health (SDoH) and access to the bike share program. A total of 322 photos and narrative comments from citizen science walk audits developed deductive themes and follow up focus groups informed inductive themes. Themes addressed challenges to access, maintenance, safety in bike transit, comfort, and environment that create barriers to use and increase inequities for lower income and historically underrepresented communities. The use of OV provided photograph, narrative, and geocoded photo location. This novel approach served as an effective tool for community action with city decision makers. The narrative research identified the impact of the barriers, and the photographs and geocoding provided clear descriptions for locations to prioritize change by adding street signs for access and safety, fixing road safety issues or bike maintenance concerns. It actively engaged the community with the city to drive discussions and plans for change in repair systems and infrastructure that also addressed equity and acknowledged the SDoH supporting residents in lower income or historically underrepresented communities. Citizen science engaged community voices, supporting change in city policies and transportation initiatives to support the sustainability of the bike share program.

## 1. Introduction/Background and Significance

Social determinants of health (SDoH), as defined by the US *Healthy People 2030* national objectives, are the “conditions in the environments where people are born, live, learn, work, play, worship, and age that affect a wide range of health, functioning, and quality-of-life outcomes and risks” [1]. Access to transportation is an important component of the built environment and a SDoH, which directly and indirectly impacts public health and health equity. In 2022, 13 to 14 million adults in the United States reported a consistent lack of reliable transportation. Individuals who reported lower education and lower family income levels had an increased likelihood of experiencing lack of reliable transportation [2]. While well-designed public transportation systems can increase access to worksites and essential resources, 45% of individuals living in disadvantaged communities still lack access to public transportation as a result of increases in travel cost and distances [2,3,4].

Shared micromobility can support utility transit. This includes the use of a bike share program or other publicly available shared mobility as a means of transportation versus the use for physical exercise or leisure activity. Shared mobility as a source of transportation has experienced significant growth over the past decade across the United States and Canada. In both countries in 2022, there were 130 million trips taken, with 71.5 million (55%) bike share trips demonstrating the highest usage. Of these 71.5 million bike share trips, 67 million were taken on station-based bike share systems and 4.5 million were taken on dockless e-bikes [5]. Since 2010, people have taken more than 730 million trips on shared micromobility in the United States and Canada [5]. Environmental factors are essential in the access and success of bike share programs for all individuals. Research by Macioszek et al. assessed the impact of random identified macro-environmental factors and their mutual interaction on bike share programs in Polish cities [6]. The study found that sustainability and growth of bike share programs was influenced not only by the structure of the bike share program but also by positive and negative aspects of the environment, including social factors (e.g., healthy life style), technological factors (e.g., frequency of dock station servicing), economic factors (e.g., free or low cost for bike use), environmental factors (e.g., bike share eco-friendliness), and political factors (e.g., promoting bike prioritized roads, promoting bike use education) [6].

Bike share programs provide a method of transportation with the potential to increase access for individuals who face barriers to personal or public modes of transportation, expanding mobility options for lower-income community members [7,8]. Though bike share programs have expanded significantly over the past decade, research has focused on the disparities in bike share use in lower-income and underrepresented ethnic minority communities. Data from bike share docking stations in lower-income areas demonstrated fewer trips compared to docking stations in higher-income areas [9]. One contributing factor is lack of equity in bike share infrastructure across cities and communities with a greater number of bike share stations in higher-income areas and business districts compared to lower-income areas. Unevenly distributed bike share infrastructure limits the availability and access to bike share stations located in lower-income areas and in diverse communities [10,11,12,13,14,15]. Community members reported barriers associated with limited bike share access in lower-income communities, including the need for additional bike share stations and bikes for use in transit, as well as the need for better bike infrastructure routes reducing biking distance to reach travel destinations [11,15,16]. Additional barriers to use included the cost of bike share use for single trips and bike share memberships [10,17,18], as well as the need for smart phone technology, the method of payment requiring a credit card, and limited awareness of education about the bike share system [17,18,19].

Safety and comfort also play critical roles in bike share program usage [11,14,20]. When the physical environment is perceived to be unsafe for cycling it can be a barrier to usage [11,14,19]. Personal safety and sharing the road with vehicle traffic, congested traffic, or poor road conditions were universal community member cycling concerns impacting on bike share usage regardless of ethnicity or economic status [21]. When biking infrastructure is lacking, cyclists are more likely to ride on the sidewalks of high-traffic roads, due to personal safety concerns, which increases their risk of violating ordinances and receiving a ticket or citation [22]. Black/African American and Hispanic/Latino cyclists, experience greater concerns of racial profiling, as evidenced by disproportionate policing and the number of cycling citations, including riding on sidewalks [22,23,24]. In Hispanic/Latino and Black/African American communities, negative factors to bike share usage included personal security concerns, such as theft, violent crime, drug dealing, or police harassment [20]. The concerns of Hispanic/Latino community members focused on being a victim of crime while biking; while the concerns of Black/African American community members focused on being stopped by police while biking [21]. Additional barriers to bike share use by Hispanic/Latino and Black/African American community members included the stigma of poverty, using the bike share out of necessity, or associating the bike share with “whiteness” and concerns of negative reaction by family members [25].

Research has also identified factors supporting bike share use in lower-income and diverse communities. Individual factors associated with increased bike share use included lower-income status, lack of access to a car, belief that bike transit provided efficiency of travel around town, or reduction of transportation costs [17,26,27]. Reasons for bike share use also included social activities for fun, exercise, as well as utility transit for school, job, and skills training [18]. Intermittent high bike share use, with more than 20 trips in a month, was associated with lower-income status and limited transportation options compared to regular bike share use [17]. Research by Ogilvie et al. found that individuals from lower-income areas on average made more trips compared to individuals from higher-income areas and were less likely to reside close to bike share stations and less likely to purchase annual memberships. However, when comparing all individual users from lower-income areas, those with annual access had higher average usage compared to those who did not [15]. Research has also highlighted bike share use among diverse communities. Community members residing in communities with a greater concentration of racial and underrepresented ethnic minority groups, and lower socio-economic status demonstrated higher bike share use at differing times of day [27]. Additionally, individual and neighborhood factors measured in the socioeconomic disadvantage index (SDI) were associated with a 9% higher level of bike share use, meaning increased socioeconomic disadvantage was associated with higher bike share use [28]. There is a growing body of research assessing strategies to reduce barriers to bike share use addressing cost with discounts, including short-term and discounted memberships, single use and reduced single use passes, and free transfers between public transit and bike share stations [9,10,29]. Additional strategies to increase access for lower-income users include use of cash or vouchers instead of a credit card [9,29]. Though studies have identified increased access, the disparities in bike share use are still evident and additional steps need to be explored to increase access for individuals experiencing barriers to use.

Community-based participatory action research (CPBAR) methods, such as photovoice and Our Voice (OV), provide mechanisms for reflective qualitative research, engaging community members as citizen scientists [30,31,32]. These qualitative visual research methodologies use photograph images, narrative explanations and, at times, provide image geolocation collected by citizen scientists focused on promoting social change. Research is limited on the use of photovoice methodology as a qualitative method for cycling and bike share use. Published research using photovoice or OV on bike share programs is not evident. Photovoice research has investigated utility cycling assessing challenges and benefits of commuter cycling. Bhandal and Noonan used photovoice with fifteen community members in Liverpool, England, using personal bikes for commuting purposes [33]. Participants identified bike commuter themes of “safety” and “comfort” as challenges and “enjoyment” and “convenience” as benefits [33]. While Guell and Ogilvie, employed photovoice with nineteen community participants using personal bikes for commuting [34]. Participants identified the benefit of personal bike commuting to be “a positive sense of wellbeing” and the challenge of personal bike commuting to be a “lack of choice due to limited transportation options” [34]. This gap in the literature provides a rich opportunity to use the CBPAR OV approach to explore SDoH through investigation of the bike share facilitators and barriers with the community.

In this paper, we aim to address this gap in the literature with the use of the OV citizen science method with community member citizen scientists to identify both positive and negative aspects of our first pilot bike share program in Fort Smith, Arkansas. We addressed the facilitators and barriers of bike share use considering SDoH on access and use for individuals from lower-income neighborhoods and individuals from underrepresented ethnically diverse communities. This research represents the continued collaborative progress towards the equity goals for our local community. In 2020, the city of Fort Smith, Arkansas participated with western Arkansas counties in the National Association of Chronic Disease Directors (NACDD) Walkability Institute supported by the Centers for Disease Control (CDC). The resulting Institute Action Plan addressed four areas of concern: (1) equitable access to mobility options; (2) ensure marginalized and diverse community leadership in transportation infrastructure investments; (3) track health and wellness outcomes from infrastructure improvements; and (4) gain citizen engagement with “OV” CBPR. We have completed several citizen science projects focused on walkability with walk audits to address access and infrastructure using the OV Discovery Tool [35]. With our community citizen scientists and the use of deductive and inductive analysis, we identified concerns surrounding access to bike share programs, limitations in technology literacy, and cost. Additional issues focused on safety and biking infrastructure, as well as concerns with the sustainability of the bike share program due to maintenance concerns. The OV photos and narrative data along with geocoded locations gathered from this project provided rich information to better understand barriers to usage and to act with the city to further address concerns, thereby improving equitable access. It also provided data to prioritize interventions and support city planning for biking infrastructure projects with clearer focus on equity. The organization of this paper is as follows: Section 1 reviews past research on bike share use and barriers to use in lower-income and underrepresented communities. It also introduces photovoice research and gaps in photovoice and OV bike share research. Section 2 identifies the qualitative research inquiry structure, the OV technology, participants, informed consent process, and qualitative analysis. Section 3 presents the results, including demographic information, as well as deductive and inductive analysis. In Section 4, the results are provided and interpreted. Section 5 provides the conclusions and remarks for the next steps in our community and the broader implications for use of the OV Discovery Tool in the bike share program as a mechanism to engage community with city government and planning in efforts to reduce some SDoH by addressing equity access biking as a form of transit.

## 2. Materials and Methods

This project was a part of a larger National Science Foundation Civic Innovation grant [36] that assessed the impacts of shared micromobility focused on increased access to jobs and essential services for diverse groups in a socioeconomically diverse mid-sized U.S. community. In the fall of 2021, 8 bike stations and 40 bikes/e-bikes were installed in Fort Smith, Arkansas by Tandem Mobility [37]. Tandem Mobility has a history of working with community driven projects and provides a turnkey bike share service, including hardware, software operations, and customer support. Following email sign up, the bike share program offered riders free thirty-minute bike share use with a fifty cent cost for each thirty minute block thereafter. The project explored the facilitators and barriers to the community bike share program focusing on three highly used stations of the 8 bike share stations.

**Figure 1:**
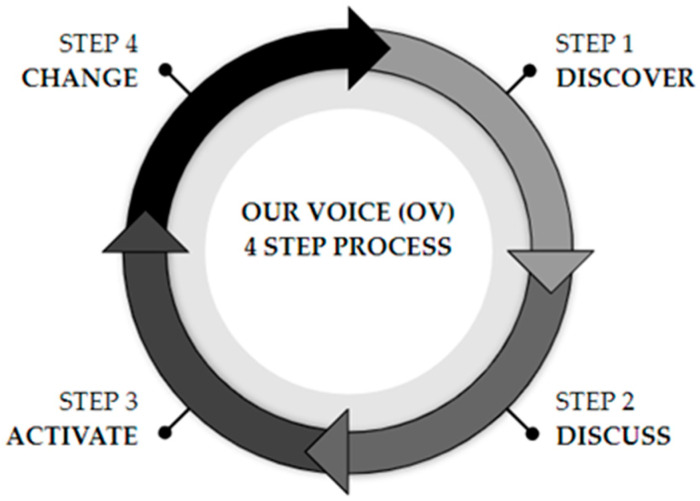
The Our Voice 4 Step Process [38].

A community-based participatory action research (CBPAR) framework and the evidence-based Our Voice (OV) four-step method guided this institutional review board approved citizen science qualitative project (Figure 1) [38]. The OV method has been demonstrated to be effective in guiding and driving health equity projects and local community change. The OV method includes active community participation across all steps of the research processes [38]. Community participants served as citizen scientists and were trained to use the OV Discovery mobile application for data collection [39]. This project explored the citizen experience related to three separate bike stations and the surrounding neighborhood environments. In addition, preclinical (yrs. 1–2) osteopathic medical students were embedded as participant researchers alongside community members. The students participated as citizen scientists in all aspects of the study. They were also tasked with assessing the benefits and barriers of the bike share program while considering the impact of social determinants of health on vulnerable individuals. Prior to participation, the medical students completed 3 didactic lectures addressing social determinants of health, health disparities, cultural competency, and cultural humility as integrated components of their osteopathic medical school preclinical year 1 and year 2 curriculum.

Following the recruitment and research training of preclinical osteopathic medical students, recruitment for this project was completed with the community in the fall of 2022. Several recruitment mechanisms were used to ensure information was inclusive and available to diverse historically underrepresented members of the community. Printed information pamphlets in English and Spanish were displayed in public libraries, grocery stores, churches, food pantries, community shelters, health clinics, free health clinics, and community centers. These pamphlets were also emailed to individuals who had signed up through Tandem Mobility to use the Fort Smith bike share program. Three informational sessions were held in public settings to discuss and answer questions about the bike share program, community research project, and the citizen scientist approach. During the recruitment period, participants were also informed of the project being voluntary and gave informed consent. Meals, snacks, and bottled water were provided for each session. Up to 45 participants were recruited for three walks to maintain 6–12 participants for each walk with osteopathic medical student researcher participation. Community members 18 years or older were eligible to participate in the project following completion of informed consent and simple training in the OV process and mobile application. Informed consent, approved by Arkansas Colleges of Health Education Institutional Review Board, was obtained from all participants involved in the project. Full project participation required three components: (1) survey(s); (2) community walk; and (3) facilitated focus group. At the end of the project, participants received a $150 gift card as compensation for their participation in the project.

All participants completed a six-item online multiple-choice demographics survey to provide information on age, gender, race/ethnicity, residential living environment, use of shared micromobility, and bike ownership.

The first OV step, “*Discover*”, was completed through citizen science walk audits. Participants completed a thirty minute to sixty minute-group community walk at one of the three identified bike share stations. To ensure safety and comfort, and to address any questions or concerns in the research process, the primary investigator (PI) was present during each walk. Medical student researchers were also present and participated during each walk. Using the OV Discovery Tool mobile application, participants captured photographs, rated the photographs as positive, negative, or neutral, and provided typed or oral explanations for each photograph taken. Each captured photograph subsequently recorded the location of where the photo narrative was documented. Wi-Fi access was required to complete the online surveys deployed through an online survey platform, to download the OV Discovery Tool application, and to upload the walk images taken at the end of a group walk. To increase comfort and accessibility, the research team provided eight cell phones with the survey downloaded, the OV Discovery Tool application installed, and a unique walk ID entered for participant use during each walk. Participants were also free to use their personal phone and download the Discovery Tool app, but personal phones had to have Wi-Fi access. Each participant walk had a unique code sent by the OV team to maintain the integrity of images, ratings, and narratives collected by citizen scientists. As per the standard OV data use protocol, all OV Discovery Tool data were collected in an anonymized fashion prior to uploading to Stanford University’s secure server for basic data cleaning and integration purposes.

Participants were directed to use the following question to guide their participation or “discovery” in the project as citizen scientists: “*What makes it easy or hard to ride a bike using the bike-share program in Fort Smith, Arkansas?*” At the completion of a walk, research devices were returned and walk images were uploaded with Wi-Fi access. Participants using personal phones with Wi-Fi access uploaded images through the OV Discovery application to the Stanford University secure server where anonymous images were processed and geo-coded for walk location. Typed and oral comments were converted to text narrative explanations, and pictorial categorical ratings (positive/neutral/negative) were also geo-coded for final visual walk data images with walk maps by the OV team. The completed walk data was sent back to the research team in PDF format grouped by walk ID through a secure firewall-protected server to be interpreted by citizen scientists.

**Figure 2:**
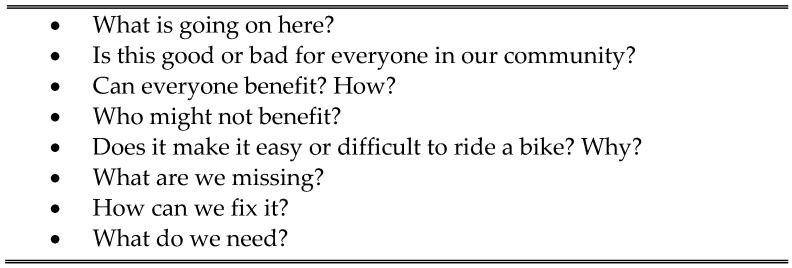
Focus Group Guiding Questions.

OV Steps two, “*Discuss*”, and three, “*Activate*”, were embedded in the focus group sessions. Citizen scientists from each walk group participated in a focus group to further explore problems and solutions as a community. Meals, snacks, and drinks were provided. Photographic images, narrative explanations, and geo-coded walk locations were printed on 210.00 mm by 297.00 mm sheets of paper for each of the three citizen science walk audits. The photographs from each walk were grouped by the deductive coding categories and each photo group was prepared for the follow-up focus group. During each focus group, participants were provided with instructions to explore each group of photographs, writing down observations on 76.20 mm by 127.00 mm inch sticky note pads. Each photo group had two large paper flip charts for observations. Participants placed individual notes on the appropriate flip chart or wrote directly on the chart. Guided discussion occurred naturally throughout the focus group session. Focus group questions were provided on 210.00 mm by 297.00 mm sheets at each table to guide participant table organic discussion, but written responses for each question were not required (Figure 2).

This project used descriptive frequencies and qualitative analysis. Both the deductive coding following walk audits and the inductive coding following facilitated focus group sessions were completed by the four osteopathic medical students who were embedded as citizen scientist researchers. Students received training from the PI, a trained qualitative researcher, who guided the process. Demographics were reported through the use of descriptive frequencies of categorical response data from the six-question survey. Deductive coding was used to categorize the citizen scientist’s walk data and was used to establish the credibility and trustworthiness of the qualitative inquiry. Deductive codes were created from the published literature and applied to data during analysis to organize data into predetermined categories [40]. In the current study, three deductive codes were used: (1) Safety [41]; (2) Access [42]; and (3) Comfort [43]. These codes were identified from the published bike share literature addressing key aspects of the guiding question, “*What makes it easy or hard to ride a bike using the bike-share program in Fort Smith?*”

All steps of analysis were guided and reviewed by the PI. All three citizen science walk audits used the same three deductive codes. The pictures collected from each walk were kept separate for coding and categorization to support trustworthiness through credibility and consistency [44]. Deductive codes were applied to the pictures and narrative explanations by each medical student researcher. The data was triangulated through multiple review meetings with student researchers and the PI. Discrepancies in coding were addressed through collaborative discussion with final group identification of a single code or consensus that an image was appropriate for the code, multiple codes, or a new code. The images from each of the three walks were then organized by the deductive code and provided to each walk focus group. The facilitated focus groups used guiding questions (Figure 2) to review and discuss the images grouped by deductive codes. Participants reviewed images and wrote narrative explanations addressing guiding questions, solutions, and concerns. The narrative data from the focus groups was used for deeper exploration into the meaning of data through the use of inductive analysis provided authenticity and rigor to the study. Focus group narrative data were organized and used to identify patterns, categories, and emerging themes [44,45]. Inductive codes were then created to further delineate participants’ lived experience as they explored the accessibility of the bike share program. The written notes and flip charts for each focus group were reviewed by each student researcher to identify patterns. Student researchers added summary notes and observations. The PI documented narrative notes from the focus group members and student researchers in an Excel file for each walk to provide clear organized narrative data. These steps were reviewed by student researchers to verify authenticity. Next, codes and patterns were vetted by each student independently. The students and PI further vetted and triangulated data through three separate review meetings focusing first on each walk and next on exploring data across all three walks. Final emerging themes and categories were established.

## 3. Results

Of the 45 community participants recruited, 27 (60.00%) participated as citizen scientists. Total participants included 27 community members and four osteopathic medical students. Of the medical students, one student was in 1st year and three were in their 2nd year of their medical school curriculum. The 27 community members provided adequate numbers for walks and focus group participation which targeted between 18 and 36 participants [46]. Of the 31 total participants, 24 (77.41%) were between 18 and 34 years of age, two (6.45%) were greater than 50 years of age, and five (16.14%) chose not to disclose their age. Male and female participation was equal, with 13 (41.94%) male participants and 13 (41.94%) female participants in each group. Five participants preferred not to disclose their gender. When considering the residential living environment, eight (26.00%) owned their place of residence, eighteen (58.00%) rented, and five (16.14%) chose not to disclose their residential living environment. When asked about ethnicity, thirteen (41.93%) identified as white, and fourteen (45.17%) identified as a minority status, with four Hispanic or Latino, four American Indian or Alaska Native, four Asian or Asian American, two black or African American. Finally, four (12.90%) chose not to disclose their ethnicity. All participants had used the bike share program at least once prior to participating in this study. When asked about bike ownership, 17 (54.83%) did not currently own or had never owned a bicycle.

A total of 325 images with narrative explanations and ratings were taken over the three-citizen science bike share neighborhood walks. Three were lost due to technical transfer issues, leaving 322 geo-coded photographs complete with narrative descriptions and geo-coded ratings. The 322 photographs were grouped as follows: Walk 1 included (156; 48.45%) photos; Walk 2 included (104; 32.30%) photos; and Walk 3 included (62; 19.25%) photos from three different citizen science walks. Of the 27, 11 (40.7%) community participants did not have access to a cell phone or to Wi-Fi and chose to use project phones for participation as citizen scientists.

Deductive analysis assessed the 322 images, narratives, and ratings by walk (Table 1). When walks 1–3 were combined, analysis supported the three deductive research based coded themes (Safety, Access, and Comfort). Of the 322 photographs, Safety represented 140 photographs (43.48%), “Access” represented 97 (30.12%), and “Comfort” represented 35 (10.87%); two additional themes were identified: “Maintenance” represented 30 photographs (9.32%) across all walks; and “Environment” represented 20 (6.21%), identified only in walk 1 and 2 data (Figure S1). When considering the impact of the themes for each walk, three of the themes (Safety, Access, and Maintenance) had greater focus on negative ratings or opportunities for change, while two themes (Comfort and Environment), focused on positive ratings or strengths. “Safety” had 66.00%, 63.00%, and 69.00% coded as negative by citizen scientists for walk sites 1–3, respectively. “Access” had negative ratings of 52.00%, 42.00%, and 50.00% for each walk audit, respectively. Maintenance had a greater focus on negative ratings, with 56.00%, 78.00%, and 75.00%, respectively. “Comfort” had positive ratings for two walks with 62.00%, 50.00%. Walk site 3 contained only four photographs with 50.00% rating, and both were positive and negative. “Environment” had greater focus on positive ratings, with 75.00% and 87.50%.

**Figure 3:**
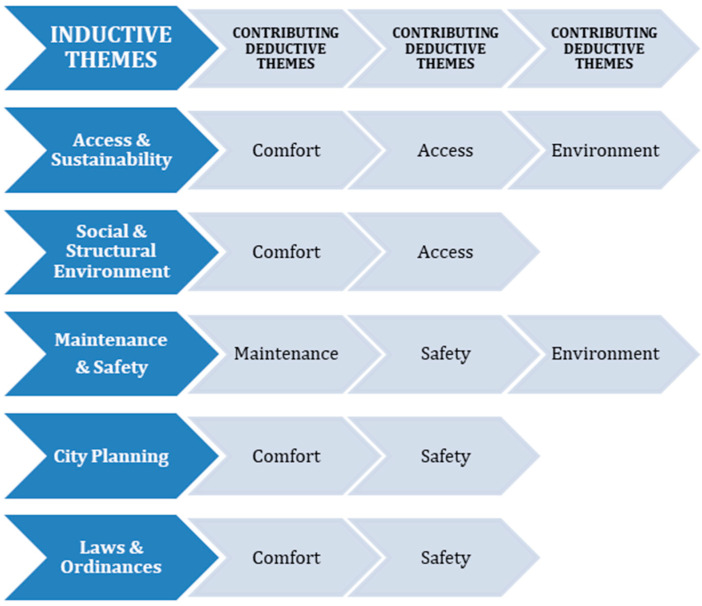
Interconnection of Deductive and Inductive Themes.

Inductive analysis of citizen science focus group participant reflection, guided discussion, and written observations of the walk image data identified five themes that embedded SDoH. These themes expanded on the deductive coded themes (Figure 3) as follows: (1) *Bike share Access and Sustainability*: Focused on technology, ease of use, technology literacy barriers, and language barriers. It also addressed concerns with bike access and bike recovery to ensure reliable transportation. (2) *Social and Structural Environment*: Incorporated accessibility topics, comfort, and the importance of developing a supportive infrastructure. In addition, convenience and availability of bikes were addressed with the need for additional bike rack stations. This would increase rider access to essential services and increase reliable transportation for ride destinations. Final concerns focused on ease of use accessing bike locking systems and the cost of the bike share program. (3) *Maintenance and Safety*: Addressed the environment, including the condition of sidewalks, streets, and green space/parks. This theme also addressed bike repair and function (charging for e-bikes) and concerns with accessible Wi-Fi and limitations in the usability of the bike share app due to slow or weak Wi-Fi signals. (4) *City Planning*: Focused on the need for infrastructure to increase useability and safety for bike share use. This included the need to complete sidewalks, create safe bike lanes for street transit, and add signage. Additional city planning suggestions included development of path connectivity, increased safety, American Disabilities Act (ADA) crosswalks [47], crosswalk lighting, and flashing lights (pedestrian light-controlled crossing) for inclusive biking access. (5) *Laws and Policies*: Addressed concerns with current city ordinances banning or limiting bike travel on city sidewalks. It also addressed concerns of the need to reduce and enforce speed limits on neighborhood streets where cars and bikes shared the road (Figure S2).

## 4. Discussion

In this project, the citizen science OV four step method was used to engage diverse community member participation in data collection and discussion, exploring facilitators and the barriers for using a bike share program for micromobility in Fort Smith, Arkansas. Overall, the project included the participation of community members from diverse ethnic backgrounds, socioeconomic levels, and bike ownership status. Thirty-one citizen scientists (twenty-seven community participants and four osteopathic medical students) actively engaged in the project using the OV Discovery Tool. The provision of project smart phones as a resource was an important factor in supporting inclusive community involvement, with 11 of the 27 (41.00%) community participants using project phones for participation. We found that using three groups for the citizen science walks provided rich opportunities to engage in the OV “Discovery” step. The sample size of 27 was adequate for the 3 walks and focus groups, meeting established qualitative group size guidance which ranges from 4 to 12 participants per group [48,49]. The follow up focus groups provided sufficient data to identify saturation for coded themes in deductive and inductive analysis respectively. Our findings are in line with previous qualitative research that has also found that rich data and identification of new themes can be accomplished without requiring a large number of focus groups [46]. For instance, Hennink et al. 2019, have indicated that the first focus group can often establish 60% of all new codes with additional focus groups providing substantially less additional data [50]. Another study found code saturation was reached with between three and six focus groups [51].

In our deductive analysis of the data from the three citizen science walk groups, we began with three research driven codes (Safety, Access, and Comfort). These codes represented approximately 85.00% of data across all three citizen science walks. Two additional codes (Maintenance and Environment) were identified, representing 15.00% of the total walk data. It was determined that the additional two codes stood out as unique from the original three codes. We returned to the literature and identified evidence supporting these codes. Maintenance focused on the impact of broken bikes on bike share usability and issues maintaining bikes serve as barriers to user satisfaction and system efficiency [52,53]. Environment can also be identified more broadly as a built environment. This includes land use, transportation systems available, and urban design with each of these aspects impacting bike share usage [5]. The impact of land use or amenities in the built environment creates lower stress routes of cycling travel highlighting the importance of non-transport aspects for cyclists and bike share users [54].

Deductive coding provided an effective framework to categorize 322 images derived from the citizen science walk audits within and across the focus groups. The framework also supported the OV “Discuss” and “Activate” steps. Organizing the data into the five categorized groups allowed community participants in each focus group to view and discuss different images that addressed broad themes related to their citizen science walks. During the focus group sessions, participants appreciated the experiential nature of the data discussion community meeting and flexibility to move images to different categorized groups, if they felt the image was better represented in another group. This maintained authentic engagement from participants’ lived experience. As a whole, the process allowed participants to engage with the data on a deeper level within and across categories, increasing rich data for inductive analysis. Informal feedback from participants validated these observations. During facilitated focus group sessions, several participants initially thought they would just look for their photographs from the walk. However, they also viewed and actively engaged in discussions with all the walk audit data from their fellow citizen scientists. Other informal comments emphasized appreciation for the grouped organization of walk data. We found that many participants were initially concerned about how to work with the large number of pictures, narrative comments, and ratings. The categorized groups of walk pictures made sense to participants and made it easy to adjust pictures if needed, and to actively discuss what was making it easy or hard to use the bike share program.

Our inductive analysis integrated the focus group responses from the walk data. Analysis identified five rich themes that expanded the original deductive walk data codes. All themes addressed current barriers and opportunities to reduce the negative impact of SDoH through the bike share program. Access to use the bikes required technology resources and technology literacy. Individuals not only need access to a smart phone with Wi-Fi but also the knowledge to successfully address challenges when navigating both the app download and the membership sign up requirements. It was noted that written instructions were only in English and assumed a level of technology literacy. Additional concerns included the need for an email address, credit card, or online bank card to sign up. This again assumed a level of financial stability that served as an invisible cost barrier to free 30 min bike access. The literature supports these findings, with McNeil et al. (2017b) reporting under-represented minority groups expressing greater concern about cost and having limited knowledge or literacy about the bike share programs [18]. Similarly, Bateman et al. (2021) identified a need for education and information about cost, procedural steps for bike share use, and safety to address barriers for potential bike share users [19]. We found it interesting that our findings also aligned with the macro-environmental perspectives provided by Macioszek et al. [6]. Our Access theme represented many of the economic factors in the environment that negatively impact both the community and the growth of bike share programs. Strategies to reduce the cost burden, such as different models to provide income-based rider discounts, have shown promise in ridership access for lower-income individuals [9,10,26]. The City of Fort Smith is working with two newly developed sectors, the City Community Mobility Department and the Inclusive Western Arkansas Leadership, to investigate different strategies through townhall and community meetings. Some strategies that have come out of these collaborative sessions include business sponsorships of bike stations, easier transfer of passes from the bike share program to public bus transit, and the creation of vouchers or pass cards as an alternative to required credit card use. The city is also investigating options for more inclusive signage with pictorial image instructions to provide a universal resource of bike share directions at stations. As with many community initiatives, place and context are critical to community member awareness. As an early component of the National Science Foundation grant, the city and community project research partners initiated community engagement, education, and biking skill safety sessions in early 2021. These sessions were held in public libraries, community centers, and in downtown business districts. Lessons learned from these activities are that individuals who have the greatest need lack the time, resources, or awareness of the programming to participate. Following this project, continued plans for additional outreach will support the transition of the project from identifying themes to engaging the community in solutions. There were concerns of the bike share’s ability to be sustainable, safe, and convenient among the focus groups. These addressed challenges in ensuring bikes were recovered and returned to stations, and that bikes were well maintained. In addition to this, there were not enough bike share racks, and the racks needed to be accessible in various service destinations, such as stores, libraries, community centers, and businesses in different neighborhoods. If bikes are not available, in disrepair, or if riders cannot locate a rack to secure the bike, the bike share fails to provide a reliable, safe resource for transportation. Other bike share programs have identified and focused on equity-related concerns, such as establishing bike stations in diverse or lower-income neighborhoods. However, community engagement and involvement are key factors in developing sustainable bike share plans in underserved communities [55]. These concerns were similar to Macioszec’s technological factors [6] including availability and usability of bikes at bike docking stations. This has been a growth process for our pilot bike share project and the challenges observed were accurately represented by participants. These barriers to use can have a greater impact on individuals with fewer resources. Research has identified that individuals from lower-income areas travel farther to access bikes [15] and may be more reliant on bike use due to lack of access to personal transportation [17]. Improvements with bike maintenance and consistency in bike recovery have been addressed with a multipronged approach. The city has created, established, and funded a Director of Community Mobility position, housed in the City official offices that oversees the bike share program, mobility activities, and champions project support of mobility infrastructure. In addition, the city has partnered with a local bicycle shop to support maintenance issues; finally, Tandem Mobility facilitates tracking of bike location for bike recovery. The expansion of bike share stations to neighborhood communities will require further assessment and city engagement. However, diverse community member involvement, and the diversity of membership in the Community Mobility Department and the Inclusive Western Arkansas Leadership will be an asset, as they are actively engaged in policy work and future infrastructure funding. When considering the environment there were concerns that addressed both comfort and safety, such as the availability of bike repair stations or lighting on trails and sidewalks. Other concerns addressed city infrastructure and planning including incomplete sidewalks, lack of sidewalks, or sidewalks in disrepair, which made it difficult to use the bike share program for transportation and were safety risks for riders. Additional infrastructure concerns noted the lack of curb cuts or safe transitions from the street for bikes or pedestrians impacting both safety and access for community members. In 2022, Schneider et al. found that in Milwaukee, WI, community members from lower-income neighborhoods, regardless of ethnicity, identified greater concerns for traffic and personal safety for individuals riding bicycles [20]. City planning was also addressed with the need for protected bike lanes that were buffered from cars. There were concerns about safety when bikes shared the road with cars and the speed limits were too high. This was an even greater concern in some areas where there were ordinances that prevented individuals from riding a bike on the sidewalks, which corroborates the previous literature. Bateman et al. found infrastructure and safety to be primary barrier concerns for community participation in bike share programs, with the lack of exclusive bike lanes identified as a primary concern [19]. Additional studies have identified concerns with safety and comfort negatively impacting access and usage of bike share programs by the community [7,11].

These concerns address macro-environmental social and political factors from research by Macioszek et al., impacting bike share sustainability [6]. McNeil et al. found that social activities and exercise, as well as utility transit positively influenced bike share use for lower-income individuals [11]. However, political factors, such as issues with safe bike transit due to lack of biking infrastructure, pedestrian-only sidewalks, and laws for vehicle speed limits, can also negatively impact social factors and ultimately reduce bike share usage. When considering shared sidewalks, pedestrians and cyclists tolerate it but it is not desirable for either, increasing the risk of conflict, accidents, and general discomfort [56]. Due to increased awareness influenced by projects like the bike share program, there are also political factors positively impacting its sustainability and future growth. The Director of Community Mobility continues to champion safe cycling, cycling path connectivity, and cycling infrastructure for shared vehicle and bike transit. These efforts have gained traction with the Department and the Inclusive Western Arkansas Leadership to address future biking infrastructure projects and funding. Additional steps have been taken to address cyclists and pedestrian concerns of safety and comfort. The City of Fort Smith has partnered with the Arkansas State Highway Safety Office on an initiative to prioritize cyclists and pedestrians that began in the fall of 2024. One aspect of this initiative is the development of a safety plan that identifies the location and frequency of accidents and prioritizes projects focused on accident mitigation. As conducted in previous OV projects with the city, the 322 OV photograph images with comments and geocoding will be provided to the city to provide additional clear community perspectives with image and location on safety risks for cyclists. This can provide insight to provide intervention or prevention to prioritize enhanced infrastructure for cyclist and pedestrian safety. One of the limitations of this qualitative project was the fact that it represents a single community experience and used only four osteopathic medical students. Study results should be interpreted within the framework of the qualitative approach including consideration of the small size of this project.

## 5. Conclusions

This NSF research project provides a novel approach to bike share evaluation through the use of the OV Discovery Tool with geocoded photographs and narratives to accompany focus group driven qualitative thematic analysis addressing facilitators and challenges of a pilot bike share program. This dual aspect of the project provides a greater level of utility. The five deductive themes (safety; access; comfort; maintenance; and environment) that further emerged to the five inductive themes (access and sustainability; social and structural environment; maintenance and safety; city planning; and laws and ordinances) offer clear narratives of strengths and concerns supporting each theme. These themes and narratives drive understanding. However, linking themes to photographs with geolocations moves beyond understanding by providing visual images and locations as tools for action by city officials. This project was developed and completed with strong community partnerships. Western Arkansas Planning and Development District (WAPDD) [57] has served as an active and engaged community partner in the present project, as well as two previous OV community walkability citizen science projects supporting OV steps 3, “Activate”, and 4, “Change”. As with previous OV projects, data and citizen science findings will be provided to WAPDD to be reviewed for future discussion and city planning. This project supports previous findings that shared micromobility has the potential to increase access to utility transit as one approach to addressing some aspects of SDoH and moving toward equity for lower-income and underrepresented minority communities. Citizen science effectively engaged the community in voicing the facilitators and barriers of the bike share to drive change in city policies and transportation initiatives. Consistent themes across all analyses identified the need to increase “access” and “safety” as clear priorities. Inductive themes provided authenticity and rigor to this study and directed a framework inclusive of community voice for policy and city discussions. In addition, the focus on access and opportunity for vulnerable members of the community to benefit from the bike share program was addressed, increasing awareness and providing challenges and opportunities to address SDoH through effective bike share access. Finally, this project was originally designed as a participatory research project to understand the impact of a bike share program. It is important to remember that goals and barriers for social and policy change need to be defined and communicated to maintain honesty and integrity for participants regarding expectations for immediate change.

## Figures and Tables

**Table 1:** Deductive Analysis Citizen Science by Walk Site.

**Walk Site 1** (represented 156 of 322 total photograph images)				
Deductive Code	Total Images	Positive (+)	Negative (−)	Both (+/−)
Access	46.00	15.00	24.00	07.00
Comfort	13.00	08.00	02.00	03.00
Maintenance	09.00	03.00	05.00	01.00
Safety	76.00	20.00	50.00	06.00
Environment	12.00	09.00	02.00	01.00
**Walk Site 2** (represented 104 of 322 total photograph images of 322)				
Deductive Code	Total Images	Positive (+)	Negative (−)	Both (+/−)
Access	31.00	15.00	13.00	04.00
Comfort	18.00	09.00	06.00	03.00
Maintenance	09.00	00.00	07.00	02.00
Safety	38.00	07.00	24.00	07.00
Environment	08.00	07.00	00.00	01.00
**Walk Site 3** (represented 62 of 322 total photograph images)				
Deductive Code	Total Images	Positive (+)	Negative (−)	Both (+/−)
Access	20.00	09.00	10.00	01.00
Comfort	04.00	00.00	02.00	02.00
Maintenance	12.00	02.00	09.00	01.00
Safety	26.00	06.00	18.00	02.00
Environment	00.00-	00.00	00.00	00.00

## Data Availability

Data are available and will be shared upon request.

## Supplementary Materials

The following supporting information can be downloaded at: https://www.mdpi.com/article/10.3390/ijerph21111488/s1, Figure S1: Citizen Science Walks Deductive Themes with Example Quotes; Figure S2: Focus Group Inductive Themes with Example Quotes.
