# Supplementary material for: Partnering with Communities to Understand Social Determinants of Health (SDoH) Impacts on Access to Shared Micromobility
Source: Int J Environ Res Public Health. 2024 Nov 8;21(11):1488. doi: 10.3390/ijerph21111488 (PMC11593566; doi:10.3390/ijerph21111488)
Supplement: Supplementary file 1 [file ijerph-21-01488-s001.zip › ijerph-3231843-supplementary.pdf]

SUPPLEMENTAL MATERIALS

Deductive Theme 1: Safety

Negative-Areas for Improvement

There are no crosswalks, and it is unsafe to ride in the street

The speed limits are too fast, and cars drive too close to bike riders. These sections are not safe

Sidewalks end with no curb cuts to transition to the road and no crosswalk. A rider could get hit.

The bike share station entry merges to the road so bike riders need to ride into traffic

Some of the streets are narrow for cars and there is not enough room for a bike riding, even though there is a painted bike telling us to "share the road"

Positive

There are some signs letting cars know there are bikes on the road

Some of the paths are wide enough for pedestrians and bike riders

Deductive Theme 2: Access

Negative-Areas for Improvement

The sidewalk starts in the middle of block-there is no way to access it

Some paths end in the middle of block or there are gaps of dirt where one sidewalk ends and the next begins which makes it hard to use

There are no bike racks where they'd be useful (neighborhoods, schools, grocery stores, free clinics, community) centers).

If you don't understand how to use the app or don't have a smart phone the bike share is not accessible; Wi-Fi can be very slow, and the app logs you out

Bikes are only available for adults no smaller sizes

Positive

Trails that are complete are easy to ride on

Instructions to download the app are visible

Deductive Theme 3: Comfort

Negative-Areas for Improvement

The bike rack sign does not let you know how to lock up a bike when you are finished riding

The buttons for some crosswalks are too close to a busy street/difficult to push when on the bike

Positive

It would be great to have the some of the bike trails go through nature; There is amazing scenery and there are scenic outlooks to rest

Where the streets are good, they are wide and smooth (evenly paved)

The bike racks are in good shape and have easy access to put the bikes up or get ready to ride

Many sidewalks and trails have good streetlights, so it is easy to ride in the evening

Deductive Theme 4: Maintenance

Negative-Areas for Improvement

Bike seats, bike chains need to be repaired, e-bike batteries are not charged

The bike baskets are not built well for storage, things (cell phone, wallet, groceries) will fall out when you are riding the bike

The roads are covered with trash and need to be cleaned

Positive

There is a nice bike fixing station on one of the trails, there should be more of these

Deductive Theme 5: Environment
Negative-Areas for Improvement
Smaller, older neighborhoods do not have bike stations
Not sure if you are allowed to ride a bike on all the sidewalks downtown
Positive
Good signage for bikes, there needs to be more
Larger, nicer neighborhoods have nice access
Nice large trees for shade as long as they are maintained and trimmed by the city
There are some nice parks and green spaces for rest and for families
Smaller, older neighborhoods do not have bike stations.

Figure S1: Citizen Science Walks Deductive Themes with Example Quotes

Inductive Theme 1: Bike-Share Access and Sustainability

Technology and Ease of Use and Bike access, Bike share Wi-Fi limitations

The directions are not clear. How do you access, use and lock bikes?

If you don't have Wi-Fi access you can't use the bikes and the Wi-Fi is not good by the bike stations, so it logs you out when you are trying to get a bike

Technology literacy barriers

We need better instructions on how to download the bikeshare app to use the bikes. If people don't use technology a lot or don't have cell phones with Wi-Fi, they can't get the bikes

Bike Recovery

Who is responsible for taking care of the bikes? There are stations without bikes, stations with too many bikes and bikes left in parks, the street

Some bikes are not returned, and e-bikes can't be recharged without someone checking on them to make sure they are at the bike station or have a charged battery

Inductive Theme 2: Social and Structural Environment

Comfort/Accessibility, Supportive Infrastructure

There is limited or no lighting on parts of the trail and it can be dangerous.

There need to be rest areas or protected areas and bike fix-it stations

Convenience/Location

There need to be more bike stations (bikes and bike racks) so people can use the bikes for transportation and dock them without worrying that the bike will be gone when they return. Stations could be placed at stores, libraries, community centers, businesses and in different neighborhoods etc.

Ease of Use/Cost

If you don't have a credit or bank card or email to create an account on the bikeshare program you cannot unlock and use the bike.

30 minutes free is great, but what if you take longer and don't have the money to pay? Not everyone has money to use the bike-share, so it doesn't help, and they need it

Inductive Theme 3: Maintenance and Safety

Environment and Bike Repair/Function

The bikes don't always work. Several bikes have ripped seats, broken bike chains, flat tires and several of the e-bikes are not charged so you can't ride them, or you are nervous about being safe or getting injured."

Sidewalks/Streets/Green Space

The sidewalks just stop...The sidewalks are not connected...There are unfinished sidewalks, cracked and uneven that go directly into busy streets and it's not safe.

Bike stations need to be by parks.

Inductive Theme 4: City Planning

Infrastructure/Path Connectivity

There is a need to plan and widen bike paths and sidewalks and make sure all are connected for bike riders and people walking. More people will be out exercising and use bikes to go shopping, visit etc.

Sidewalks need curb cuts and more crosswalks to encourage people to ride bikes by increasing safe access to bikes.

Safe Biking/Bike Lanes

Barriers and buffers are needed between cars and bikers...Bike lanes are needed to encourage biking and increase safety

Signage

Where there is a lot of car traffic, there need to be signs to let drivers know about bikes. Without this, it makes it difficult if someone is relying on the bike for transportation.

Signs are needed to let people know where the bike stations are and where people can bike safely. Flashing signs would be great to increase safety and alert others of cyclists and walkers.

Safety ADA crosswalks for inclusive biking access

Inductive Theme 5: Laws and Ordinances

Changing Ordinances

There are city ordinances in certain parts of the city where the bikeshare stations are located that limit or do not permit bikes on sidewalks. These need to be changed or safe bike paths need to be created so people can travel safely on a bike without getting in trouble or getting a fine.

Changing/Enforcing Speed Limits

Speed limits should be reduced on some streets and enforced in some neighborhoods so people know they can ride a bike safely.

Figure S2. Focus Group Inductive Themes with Example Quotes
